# Bis(acetato-κ^2^ *O*,*O*′)(2,2′:6′,2′′-terpyridine-κ^3^ *N*,*N*′,*N*′′)manganese(II) dihydrate

**DOI:** 10.1107/S1600536811055802

**Published:** 2012-01-07

**Authors:** Kwang Ha

**Affiliations:** a School of Applied Chemical Engineering, The Research Institute of Catalysis, Chonnam National University, Gwangju 500-757, Republic of Korea

## Abstract

The Mn^II^ ion in the title compound, [Mn(CH_3_CO_2_)_2_(C_15_H_11_N_3_)]·2H_2_O, is seven-coordinated in a considerably distorted penta­gonal–bipyramidal geometry by three N atoms of the tridentate 2,2′:6′,2′′-terpyridine ligand and four O atoms from two acetate anions which chelate the Mn atom *via* two O atoms. The lateral pyridine rings are slightly inclined to the central pyridine ring, making dihedral angles of 13.6 (2) and 5.7 (2)°. The complex and solvent water mol­ecules are linked by inter­molecular O—H⋯O hydrogen bonds into a three-dimensional network.

## Related literature

For the crystal structure of 2,2′:6′,2"-terpyridine (terpy), see: Bessel *et al.* (1992 ▶); Bowes *et al.* (2005 ▶). For related Mn(II)–terpy complexes, see: Baffert *et al.* (2004 ▶); Rich *et al.* (2010 ▶).
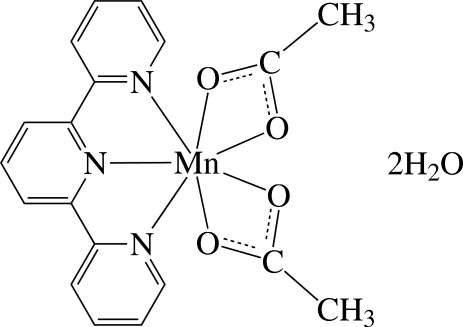


## Experimental

### 

#### Crystal data

[Mn(C_2_H_3_O_2_)_2_(C_15_H_11_N_3_)]·2H_2_O
*M* *_r_* = 442.33
Monoclinic, 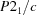

*a* = 8.4367 (10) Å
*b* = 22.921 (2) Å
*c* = 11.4952 (11) Å
β = 116.532 (7)°
*V* = 1988.8 (3) Å^3^
*Z* = 4
Mo *K*α radiation
μ = 0.71 mm^−1^
*T* = 200 K
0.38 × 0.21 × 0.20 mm

#### Data collection

Bruker SMART 1000 CCD diffractometer
Absorption correction: multi-scan (*SADABS*; Bruker, 2007 ▶) *T*_min_ = 0.770, *T*_max_ = 1.000
14786 measured reflections
4936 independent reflections
2448 reflections with *I* > 2σ(*I*)
*R*_int_ = 0.084

#### Refinement

*R*[*F* ^2^ > 2σ(*F* ^2^)] = 0.055
*wR*(*F* ^2^) = 0.146
*S* = 0.93
4936 reflections
276 parameters
4 restraints
H atoms treated by a mixture of independent and constrained refinement
Δρ_max_ = 0.69 e Å^−3^
Δρ_min_ = −0.52 e Å^−3^

### 

Data collection: *SMART* (Bruker, 2007 ▶); cell refinement: *SAINT* (Bruker, 2007 ▶); data reduction: *SAINT*; program(s) used to solve structure: *SHELXS97* (Sheldrick, 2008 ▶); program(s) used to refine structure: *SHELXL97* (Sheldrick, 2008 ▶); molecular graphics: *ORTEP-3* (Farrugia, 1997 ▶) and *PLATON* (Spek, 2009 ▶); software used to prepare material for publication: *SHELXL97*.

## Supplementary Material

Crystal structure: contains datablock(s) global. DOI: 10.1107/S1600536811055802/vn2028sup1.cif

Additional supplementary materials:  crystallographic information; 3D view; checkCIF report

## Figures and Tables

**Table 1:** Selected bond lengths (Å)

Mn1—O1	2.199 (2)
Mn1—O2	2.419 (3)
Mn1—O3	2.212 (2)
Mn1—O4	2.365 (3)
Mn1—N1	2.265 (3)
Mn1—N2	2.324 (3)
Mn1—N3	2.337 (3)

**Table 2:** Hydrogen-bond geometry (Å, °)

*D*—H⋯*A*	*D*—H	H⋯*A*	*D*⋯*A*	*D*—H⋯*A*
O5—H5*A*⋯O6^i^	0.83 (1)	2.02 (2)	2.840 (4)	168 (5)
O5—H5*B*⋯O1^ii^	0.84 (1)	1.99 (2)	2.815 (4)	168 (5)
O6—H6*A*⋯O3	0.84 (1)	2.00 (1)	2.841 (4)	174 (5)
O6—H6*B*⋯O2^ii^	0.85 (1)	2.05 (2)	2.879 (4)	168 (5)

Symmetry codes: (i) 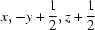; (ii) 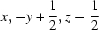.

**Fig. 1.**
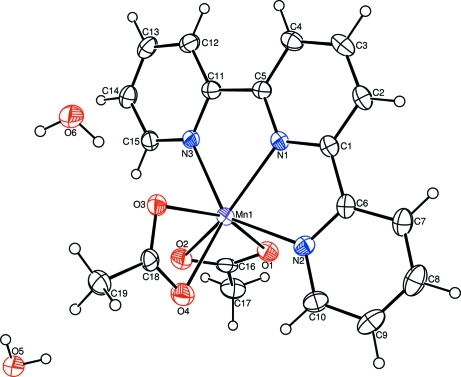
The structure of the title compound, with displacement ellipsoids drawn at the 30% probability level; H atoms are shown as small circles of arbitrary radius.

**Fig. 2.**
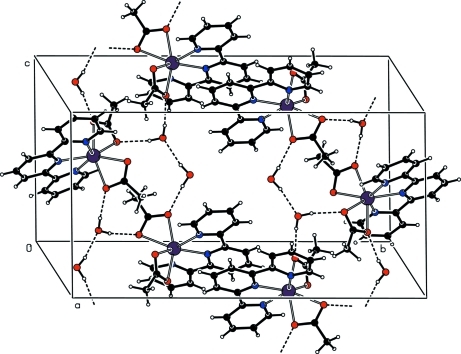
View of the unit-cell contents of the title compound. Hydrogen-bond interactions are drawn with dashed lines.

## supplementary crystallographic information

### Comment

The X-ray crystal structures of 2,2':6',2"-terpyridine (terpy) (Bessel *et al.*, 1992; Bowes *et al.*, 2005) and mononuclear Mn(II)-terpy complexes, such as [Mn(NO_3_)_2_(terpy)(H_2_O)] (Baffert *et al.*, 2004) and [Mn(C_2_F_3_O_2_)_2_(terpy)(H_2_O)] (Rich *et al.*, 2010), have been investigated previously.

The title compound consists of the neutral Mn^II^ complex [Mn(C_2_H_3_O_2_)_2_(terpy)] and two solvent water molecules. In the complex, the Mn^II^ ion is seven-coordinated in a considerably distorted pentagonal-bipyramidal geometry by three N atoms of the tridentate terpy ligand and four O atoms from two anionic acetato ligands (Fig. 1). The acetate anions chelate the Mn atom *via* two O atoms. The Mn—O and Mn—N bond lengths are somewhat different, respectively (Table 1). The Mn1—N1 (central pyridyl) bond is slightly longer than the Mn1—N2/3 (lateral pyridyl) bonds, and the Mn1—O2/4 (equatorial) bonds are somewhat longer than the Mn1—O1/3(axial) bonds. The O—Mn—O chelating angles [O1—Mn1—O2 = 56.36 (9)° and O3—Mn1—O4 = 57.04 (9)°] are considerably smaller than the N—Mn—N chelating angles [N1—Mn1—N2 = 70.52 (9)° and N1—Mn1—N3 = 69.98 (9)°] and the apical O1—Mn1—O3 angle is fairly bent with a bond angle of 158.48 (9)°. The carboxylate groups of the anionic ligands appear to be delocalized on the basis of the C—O bond lengths [C—O: 1.234 (4)–1.282 (4) Å]. In the crystal, the two lateral pyridyl rings are slightly inclined to the central pyridyl ring, making dihedral angles of 13.6 (2)° and 5.7 (2)°. The dihedral angle between the lateral pyridyl rings is 19.3 (1)°. The complex and solvent water molecules are linked by intermolecular O—H···O hydrogen bonds into a three-dimensional network (Fig. 2 and Table 2). The complex molecules stack in columns along the *a* axis and display numerous intermolecular π-π interactions between the pyridyl rings, with a shortest centroid-centroid distance of 3.773 (2) Å.

### Experimental

To a solution of Mn(CH_3_CO_2_)_2_.4H_2_O (0.1239 g, 0.506 mmol) in EtOH (30 ml) was added 2,2':6',2"-terpyridine (0.1184 g, 0.508 mmol) and stirred for 7 h at room temperature. After evaporation of the solvent, the residue was washed with acetone/ether and dried under vacuum, to give a yellow powder (0.1890 g). Crystals suitable for X-ray analysis were obtained by slow evaporation from an EtOH solution.

### Refinement

Carbon-bound H atoms were positioned geometrically and allowed to ride on their respective parent atoms [C—H = 0.95 Å (CH) or 0.98 Å (CH_3_) and *U*_iso_(H) = 1.2*U*_eq_(C) or 1.5*U*_eq_(methyl C)]. The H atoms of the water molecules were located from Fourier difference maps and allowed to refine with the restraint instruction *DFIX* (*DFIX* 0.84 0.01 O5 H5a O5 H5b O6 H6a O6 H6b) and *U*_iso_(H) = 1.5*U*_eq_(O). The highest peak (0.69 e Å^-3^) and the deepest hole (-0.52 e Å^-3^) in the difference Fourier map are located 1.04 Å and 0.85 Å from the atoms O1 and Mn1, respectively.

### Crystal data

**Table:**

[Mn(C_2_H_3_O_2_)_2_(C_15_H_11_N_3_)]·2H_2_O	*F*(000) = 916
*M**_r_* = 442.33	*D*_x_ = 1.477 Mg m^−^^3^
Monoclinic, *P*2_1_/*c*	Mo *K*α radiation, λ = 0.71073 Å
Hall symbol: -P 2ybc	Cell parameters from 2512 reflections
*a* = 8.4367 (10) Å	θ = 2.7–23.8°
*b* = 22.921 (2) Å	µ = 0.71 mm^−^^1^
*c* = 11.4952 (11) Å	*T* = 200 K
β = 116.532 (7)°	Rod, yellow
*V* = 1988.8 (3) Å^3^	0.38 × 0.21 × 0.20 mm
*Z* = 4	

### Data collection

**Table:**

Bruker SMART 1000 CCD diffractometer	4936 independent reflections
Radiation source: fine-focus sealed tube	2448 reflections with *I* > 2σ(*I*)
graphite	*R*_int_ = 0.084
φ and ω scans	θ_max_ = 28.3°, θ_min_ = 1.8°
Absorption correction: multi-scan (*SADABS*; Bruker, 2007)	*h* = −11→11
*T*_min_ = 0.770, *T*_max_ = 1.000	*k* = −30→24
14786 measured reflections	*l* = −15→9

### Refinement

**Table:**

Refinement on *F*^2^	Primary atom site location: structure-invariant direct methods
Least-squares matrix: full	Secondary atom site location: difference Fourier map
*R*[*F*^2^ > 2σ(*F*^2^)] = 0.055	Hydrogen site location: inferred from neighbouring sites
*wR*(*F*^2^) = 0.146	H atoms treated by a mixture of independent and constrained refinement
*S* = 0.93	*w* = 1/[σ^2^(*F*_o_^2^) + (0.0601*P*)^2^] where *P* = (*F*_o_^2^ + 2*F*_c_^2^)/3
4936 reflections	(Δ/σ)_max_ < 0.001
276 parameters	Δρ_max_ = 0.69 e Å^−^^3^
4 restraints	Δρ_min_ = −0.52 e Å^−^^3^

### Special details

**Table:**

Geometry. All e.s.d.'s (except the e.s.d. in the dihedral angle between two l.s. planes) are estimated using the full covariance matrix. The cell e.s.d.'s are taken into account individually in the estimation of e.s.d.'s in distances, angles and torsion angles; correlations between e.s.d.'s in cell parameters are only used when they are defined by crystal symmetry. An approximate (isotropic) treatment of cell e.s.d.'s is used for estimating e.s.d.'s involving l.s. planes.
Refinement. Refinement of *F*^2^ against ALL reflections. The weighted *R*-factor *wR* and goodness of fit *S* are based on *F*^2^, conventional *R*-factors *R* are based on *F*, with *F* set to zero for negative *F*^2^. The threshold expression of *F*^2^ > σ(*F*^2^) is used only for calculating *R*-factors(gt) *etc*. and is not relevant to the choice of reflections for refinement. *R*-factors based on *F*^2^ are statistically about twice as large as those based on *F*, and *R*- factors based on ALL data will be even larger.

### Fractional atomic coordinates and isotropic or equivalent isotropic displacement parameters (Å^2^)

**Table:**

	*x*	*y*	*z*	*U*_iso_*/*U*_eq_	
Mn1	0.26540 (7)	0.13751 (2)	0.54271 (5)	0.03290 (18)	
O1	0.1922 (3)	0.14625 (10)	0.7030 (2)	0.0428 (6)	
O2	0.1033 (4)	0.21981 (11)	0.5673 (3)	0.0560 (8)	
O3	0.3431 (3)	0.16451 (10)	0.3906 (2)	0.0405 (6)	
O4	0.4740 (4)	0.21223 (11)	0.5744 (3)	0.0582 (8)	
N1	0.2398 (3)	0.04015 (11)	0.5054 (3)	0.0312 (7)	
N2	0.5176 (4)	0.09085 (12)	0.6946 (3)	0.0375 (7)	
N3	−0.0136 (3)	0.11578 (12)	0.3744 (3)	0.0334 (7)	
C1	0.3713 (4)	0.00393 (14)	0.5827 (3)	0.0345 (8)	
C2	0.3661 (5)	−0.05520 (15)	0.5545 (4)	0.0409 (9)	
H2	0.4590	−0.0803	0.6094	0.049*	
C3	0.2266 (5)	−0.07666 (15)	0.4475 (4)	0.0436 (10)	
H3	0.2225	−0.1170	0.4275	0.052*	
C4	0.0912 (5)	−0.04049 (14)	0.3678 (4)	0.0411 (9)	
H4	−0.0063	−0.0553	0.2928	0.049*	
C5	0.1010 (4)	0.01863 (13)	0.4002 (3)	0.0314 (8)	
C6	0.5152 (4)	0.03215 (15)	0.6959 (3)	0.0373 (8)	
C7	0.6426 (5)	0.00040 (18)	0.7989 (4)	0.0507 (10)	
H7	0.6386	−0.0410	0.7992	0.061*	
C8	0.7729 (5)	0.0299 (2)	0.8991 (4)	0.0596 (12)	
H8	0.8592	0.0089	0.9705	0.071*	
C9	0.7801 (5)	0.0900 (2)	0.8973 (4)	0.0570 (12)	
H9	0.8717	0.1109	0.9658	0.068*	
C10	0.6486 (5)	0.11902 (17)	0.7917 (4)	0.0448 (9)	
H10	0.6523	0.1604	0.7887	0.054*	
C11	−0.0399 (4)	0.06118 (14)	0.3250 (3)	0.0349 (8)	
C12	−0.1923 (5)	0.04609 (16)	0.2149 (3)	0.0416 (9)	
H12	−0.2071	0.0075	0.1815	0.050*	
C13	−0.3217 (5)	0.08722 (18)	0.1541 (4)	0.0466 (10)	
H13	−0.4262	0.0776	0.0779	0.056*	
C14	−0.2971 (5)	0.14280 (17)	0.2059 (4)	0.0449 (10)	
H14	−0.3855	0.1719	0.1671	0.054*	
C15	−0.1423 (5)	0.15510 (15)	0.3145 (3)	0.0389 (9)	
H15	−0.1256	0.1935	0.3490	0.047*	
C16	0.1112 (5)	0.19443 (16)	0.6667 (4)	0.0429 (9)	
C17	0.0253 (5)	0.22051 (16)	0.7429 (4)	0.0558 (11)	
H17A	−0.0840	0.2404	0.6837	0.084*	
H17B	0.1061	0.2487	0.8053	0.084*	
H17C	−0.0027	0.1896	0.7897	0.084*	
C18	0.4454 (5)	0.20531 (15)	0.4602 (4)	0.0374 (9)	
C19	0.5335 (5)	0.24438 (16)	0.4018 (4)	0.0528 (11)	
H19A	0.4909	0.2347	0.3096	0.079*	
H19B	0.6621	0.2388	0.4475	0.079*	
H19C	0.5049	0.2851	0.4100	0.079*	
O5	0.2521 (4)	0.41065 (12)	0.4361 (3)	0.0583 (8)	
H5A	0.236 (6)	0.3863 (17)	0.484 (4)	0.087*	
H5B	0.246 (6)	0.3902 (18)	0.374 (3)	0.087*	
O6	0.1848 (5)	0.15792 (12)	0.1149 (3)	0.0694 (9)	
H6A	0.236 (6)	0.158 (2)	0.1967 (10)	0.104*	
H6B	0.152 (7)	0.1923 (9)	0.089 (5)	0.104*	

### Atomic displacement parameters (Å^2^)

**Table:**

	*U*^11^	*U*^22^	*U*^33^	*U*^12^	*U*^13^	*U*^23^
Mn1	0.0357 (3)	0.0292 (3)	0.0328 (3)	−0.0014 (2)	0.0145 (2)	−0.0005 (2)
O1	0.0457 (15)	0.0394 (15)	0.0481 (16)	0.0013 (12)	0.0253 (13)	−0.0050 (12)
O2	0.076 (2)	0.0411 (16)	0.0497 (18)	−0.0006 (14)	0.0275 (16)	−0.0045 (14)
O3	0.0393 (14)	0.0383 (14)	0.0392 (15)	−0.0004 (11)	0.0132 (12)	0.0048 (12)
O4	0.082 (2)	0.0508 (17)	0.0477 (18)	−0.0165 (14)	0.0338 (16)	−0.0040 (14)
N1	0.0332 (16)	0.0290 (15)	0.0354 (17)	0.0015 (12)	0.0188 (14)	0.0007 (12)
N2	0.0350 (17)	0.0416 (18)	0.0352 (18)	0.0021 (14)	0.0149 (14)	−0.0031 (14)
N3	0.0333 (16)	0.0337 (16)	0.0321 (16)	−0.0034 (13)	0.0136 (14)	0.0024 (13)
C1	0.034 (2)	0.0327 (19)	0.043 (2)	0.0016 (15)	0.0230 (18)	0.0042 (16)
C2	0.044 (2)	0.031 (2)	0.055 (3)	0.0070 (17)	0.030 (2)	0.0075 (17)
C3	0.055 (2)	0.0276 (19)	0.064 (3)	−0.0036 (18)	0.041 (2)	−0.0045 (18)
C4	0.047 (2)	0.035 (2)	0.050 (2)	−0.0080 (17)	0.030 (2)	−0.0063 (18)
C5	0.0319 (19)	0.0326 (19)	0.0338 (19)	−0.0038 (15)	0.0182 (16)	−0.0028 (15)
C6	0.034 (2)	0.043 (2)	0.041 (2)	0.0073 (16)	0.0216 (17)	0.0104 (17)
C7	0.042 (2)	0.057 (3)	0.051 (3)	0.0115 (19)	0.019 (2)	0.017 (2)
C8	0.037 (2)	0.089 (4)	0.050 (3)	0.016 (2)	0.017 (2)	0.019 (2)
C9	0.034 (2)	0.089 (4)	0.039 (2)	0.000 (2)	0.009 (2)	−0.005 (2)
C10	0.041 (2)	0.053 (2)	0.041 (2)	−0.0021 (18)	0.019 (2)	−0.0100 (19)
C11	0.035 (2)	0.037 (2)	0.037 (2)	−0.0056 (16)	0.0196 (17)	−0.0030 (16)
C12	0.042 (2)	0.048 (2)	0.034 (2)	−0.0098 (18)	0.0168 (18)	−0.0036 (17)
C13	0.034 (2)	0.067 (3)	0.032 (2)	−0.0053 (19)	0.0092 (18)	0.0051 (19)
C14	0.036 (2)	0.055 (3)	0.040 (2)	0.0064 (18)	0.0139 (18)	0.0117 (19)
C15	0.038 (2)	0.038 (2)	0.040 (2)	−0.0003 (16)	0.0159 (18)	0.0062 (16)
C16	0.040 (2)	0.037 (2)	0.044 (2)	−0.0121 (17)	0.0116 (19)	−0.0141 (19)
C17	0.054 (3)	0.053 (3)	0.066 (3)	0.003 (2)	0.032 (2)	−0.016 (2)
C18	0.036 (2)	0.032 (2)	0.040 (2)	0.0096 (16)	0.0138 (18)	0.0081 (17)
C19	0.054 (3)	0.049 (2)	0.056 (3)	−0.0101 (19)	0.025 (2)	0.007 (2)
O5	0.070 (2)	0.0457 (18)	0.0453 (19)	−0.0089 (14)	0.0137 (16)	0.0009 (13)
O6	0.107 (3)	0.0447 (17)	0.0449 (18)	0.0052 (17)	0.024 (2)	−0.0060 (15)

### Geometric parameters (Å, °)

**Table:**

Mn1—O1	2.199 (2)	C7—C8	1.364 (6)
Mn1—O2	2.419 (3)	C7—H7	0.9500
Mn1—O3	2.212 (2)	C8—C9	1.378 (6)
Mn1—O4	2.365 (3)	C8—H8	0.9500
Mn1—N1	2.265 (3)	C9—C10	1.395 (5)
Mn1—N2	2.324 (3)	C9—H9	0.9500
Mn1—N3	2.337 (3)	C10—H10	0.9500
O1—C16	1.267 (4)	C11—C12	1.386 (5)
O2—C16	1.257 (4)	C12—C13	1.374 (5)
O3—C18	1.282 (4)	C12—H12	0.9500
O4—C18	1.234 (4)	C13—C14	1.382 (5)
N1—C5	1.346 (4)	C13—H13	0.9500
N1—C1	1.352 (4)	C14—C15	1.374 (5)
N2—C10	1.335 (4)	C14—H14	0.9500
N2—C6	1.346 (4)	C15—H15	0.9500
N3—C15	1.341 (4)	C16—C17	1.488 (5)
N3—C11	1.351 (4)	C17—H17A	0.9800
C1—C2	1.390 (4)	C17—H17B	0.9800
C1—C6	1.474 (5)	C17—H17C	0.9800
C2—C3	1.358 (5)	C18—C19	1.500 (5)
C2—H2	0.9500	C19—H19A	0.9800
C3—C4	1.377 (5)	C19—H19B	0.9800
C3—H3	0.9500	C19—H19C	0.9800
C4—C5	1.398 (4)	O5—H5A	0.833 (10)
C4—H4	0.9500	O5—H5B	0.838 (10)
C5—C11	1.481 (4)	O6—H6A	0.842 (10)
C6—C7	1.396 (5)	O6—H6B	0.845 (10)
			
O1—Mn1—O3	158.48 (9)	C4—C5—C11	123.1 (3)
O1—Mn1—N1	102.11 (9)	N2—C6—C7	121.4 (3)
O3—Mn1—N1	99.37 (9)	N2—C6—C1	116.1 (3)
O1—Mn1—N2	85.31 (10)	C7—C6—C1	122.5 (3)
O3—Mn1—N2	103.12 (9)	C8—C7—C6	118.8 (4)
N1—Mn1—N2	70.52 (9)	C8—C7—H7	120.6
O1—Mn1—N3	99.03 (9)	C6—C7—H7	120.6
O3—Mn1—N3	87.14 (9)	C7—C8—C9	120.5 (4)
N1—Mn1—N3	69.98 (9)	C7—C8—H8	119.8
N2—Mn1—N3	140.29 (10)	C9—C8—H8	119.8
O1—Mn1—O4	105.87 (9)	C8—C9—C10	117.8 (4)
O3—Mn1—O4	57.04 (9)	C8—C9—H9	121.1
N1—Mn1—O4	138.10 (10)	C10—C9—H9	121.1
N2—Mn1—O4	81.30 (10)	N2—C10—C9	122.4 (4)
N3—Mn1—O4	133.40 (10)	N2—C10—H10	118.8
O1—Mn1—O2	56.36 (9)	C9—C10—H10	118.8
O3—Mn1—O2	104.88 (9)	N3—C11—C12	121.8 (3)
N1—Mn1—O2	141.21 (10)	N3—C11—C5	115.2 (3)
N2—Mn1—O2	130.30 (10)	C12—C11—C5	122.9 (3)
N3—Mn1—O2	81.37 (9)	C13—C12—C11	119.7 (3)
O4—Mn1—O2	80.69 (10)	C13—C12—H12	120.2
O1—Mn1—C18	132.27 (10)	C11—C12—H12	120.2
O3—Mn1—C18	29.11 (10)	C12—C13—C14	118.8 (3)
N1—Mn1—C18	122.40 (10)	C12—C13—H13	120.6
N2—Mn1—C18	93.45 (10)	C14—C13—H13	120.6
N3—Mn1—C18	110.98 (11)	C15—C14—C13	118.6 (3)
O4—Mn1—C18	27.99 (9)	C15—C14—H14	120.7
O2—Mn1—C18	91.67 (10)	C13—C14—H14	120.7
C16—O1—Mn1	96.6 (2)	N3—C15—C14	123.5 (3)
C16—O2—Mn1	86.7 (2)	N3—C15—H15	118.2
C18—O3—Mn1	93.8 (2)	C14—C15—H15	118.2
C18—O4—Mn1	88.0 (2)	O2—C16—O1	120.3 (4)
C5—N1—C1	119.6 (3)	O2—C16—C17	120.4 (4)
C5—N1—Mn1	120.2 (2)	O1—C16—C17	119.2 (4)
C1—N1—Mn1	120.0 (2)	C16—C17—H17A	109.5
C10—N2—C6	119.0 (3)	C16—C17—H17B	109.5
C10—N2—Mn1	122.9 (2)	H17A—C17—H17B	109.5
C6—N2—Mn1	117.1 (2)	C16—C17—H17C	109.5
C15—N3—C11	117.6 (3)	H17A—C17—H17C	109.5
C15—N3—Mn1	124.6 (2)	H17B—C17—H17C	109.5
C11—N3—Mn1	117.5 (2)	O4—C18—O3	121.0 (3)
N1—C1—C2	120.9 (3)	O4—C18—C19	119.9 (3)
N1—C1—C6	114.9 (3)	O3—C18—C19	119.1 (3)
C2—C1—C6	124.2 (3)	O4—C18—Mn1	64.0 (2)
C3—C2—C1	119.3 (3)	O3—C18—Mn1	57.12 (18)
C3—C2—H2	120.4	C19—C18—Mn1	174.9 (3)
C1—C2—H2	120.4	C18—C19—H19A	109.5
C2—C3—C4	120.6 (3)	C18—C19—H19B	109.5
C2—C3—H3	119.7	H19A—C19—H19B	109.5
C4—C3—H3	119.7	C18—C19—H19C	109.5
C3—C4—C5	118.3 (3)	H19A—C19—H19C	109.5
C3—C4—H4	120.9	H19B—C19—H19C	109.5
C5—C4—H4	120.9	H5A—O5—H5B	103 (5)
N1—C5—C4	121.2 (3)	H6A—O6—H6B	109 (5)
N1—C5—C11	115.6 (3)		
			
O3—Mn1—O1—C16	33.2 (4)	O2—Mn1—N3—C11	−164.4 (2)
N1—Mn1—O1—C16	−143.5 (2)	C18—Mn1—N3—C11	107.1 (2)
N2—Mn1—O1—C16	147.6 (2)	C5—N1—C1—C2	0.6 (5)
N3—Mn1—O1—C16	−72.2 (2)	Mn1—N1—C1—C2	−173.9 (2)
O4—Mn1—O1—C16	68.0 (2)	C5—N1—C1—C6	−179.3 (3)
O2—Mn1—O1—C16	1.07 (19)	Mn1—N1—C1—C6	6.2 (4)
C18—Mn1—O1—C16	57.2 (2)	N1—C1—C2—C3	0.0 (5)
O1—Mn1—O2—C16	−1.07 (19)	C6—C1—C2—C3	179.9 (3)
O3—Mn1—O2—C16	−169.4 (2)	C1—C2—C3—C4	−0.2 (5)
N1—Mn1—O2—C16	63.7 (3)	C2—C3—C4—C5	−0.2 (5)
N2—Mn1—O2—C16	−47.2 (3)	C1—N1—C5—C4	−1.0 (5)
N3—Mn1—O2—C16	105.9 (2)	Mn1—N1—C5—C4	173.5 (2)
O4—Mn1—O2—C16	−117.3 (2)	C1—N1—C5—C11	177.3 (3)
C18—Mn1—O2—C16	−143.1 (2)	Mn1—N1—C5—C11	−8.1 (4)
O1—Mn1—O3—C18	38.2 (3)	C3—C4—C5—N1	0.8 (5)
N1—Mn1—O3—C18	−145.07 (19)	C3—C4—C5—C11	−177.4 (3)
N2—Mn1—O3—C18	−73.1 (2)	C10—N2—C6—C7	2.5 (5)
N3—Mn1—O3—C18	145.76 (19)	Mn1—N2—C6—C7	−166.6 (3)
O4—Mn1—O3—C18	−2.70 (18)	C10—N2—C6—C1	−177.4 (3)
O2—Mn1—O3—C18	65.5 (2)	Mn1—N2—C6—C1	13.5 (4)
O1—Mn1—O4—C18	−162.7 (2)	N1—C1—C6—N2	−13.0 (4)
O3—Mn1—O4—C18	2.80 (19)	C2—C1—C6—N2	167.1 (3)
N1—Mn1—O4—C18	67.2 (3)	N1—C1—C6—C7	167.1 (3)
N2—Mn1—O4—C18	114.7 (2)	C2—C1—C6—C7	−12.7 (5)
N3—Mn1—O4—C18	−43.2 (3)	N2—C6—C7—C8	−0.8 (6)
O2—Mn1—O4—C18	−111.8 (2)	C1—C6—C7—C8	179.1 (4)
O1—Mn1—N1—C5	105.3 (2)	C6—C7—C8—C9	−1.1 (6)
O3—Mn1—N1—C5	−73.4 (2)	C7—C8—C9—C10	1.2 (6)
N2—Mn1—N1—C5	−174.1 (3)	C6—N2—C10—C9	−2.3 (5)
N3—Mn1—N1—C5	10.0 (2)	Mn1—N2—C10—C9	166.0 (3)
O4—Mn1—N1—C5	−123.5 (2)	C8—C9—C10—N2	0.5 (6)
O2—Mn1—N1—C5	54.9 (3)	C15—N3—C11—C12	1.3 (5)
C18—Mn1—N1—C5	−92.7 (3)	Mn1—N3—C11—C12	−171.8 (3)
O1—Mn1—N1—C1	−80.2 (2)	C15—N3—C11—C5	−176.1 (3)
O3—Mn1—N1—C1	101.1 (2)	Mn1—N3—C11—C5	10.9 (4)
N2—Mn1—N1—C1	0.4 (2)	N1—C5—C11—N3	−2.1 (4)
N3—Mn1—N1—C1	−175.5 (3)	C4—C5—C11—N3	176.2 (3)
O4—Mn1—N1—C1	51.0 (3)	N1—C5—C11—C12	−179.5 (3)
O2—Mn1—N1—C1	−130.5 (2)	C4—C5—C11—C12	−1.1 (5)
C18—Mn1—N1—C1	81.8 (3)	N3—C11—C12—C13	−0.7 (5)
O1—Mn1—N2—C10	−71.7 (3)	C5—C11—C12—C13	176.5 (3)
O3—Mn1—N2—C10	88.3 (3)	C11—C12—C13—C14	−0.7 (5)
N1—Mn1—N2—C10	−176.3 (3)	C12—C13—C14—C15	1.4 (5)
N3—Mn1—N2—C10	−170.2 (2)	C11—N3—C15—C14	−0.6 (5)
O4—Mn1—N2—C10	35.2 (3)	Mn1—N3—C15—C14	171.9 (3)
O2—Mn1—N2—C10	−34.6 (3)	C13—C14—C15—N3	−0.8 (6)
C18—Mn1—N2—C10	60.5 (3)	Mn1—O2—C16—O1	1.8 (3)
O1—Mn1—N2—C6	96.9 (2)	Mn1—O2—C16—C17	−178.0 (3)
O3—Mn1—N2—C6	−103.1 (2)	Mn1—O1—C16—O2	−2.0 (4)
N1—Mn1—N2—C6	−7.7 (2)	Mn1—O1—C16—C17	177.8 (3)
N3—Mn1—N2—C6	−1.5 (3)	Mn1—O4—C18—O3	−4.7 (3)
O4—Mn1—N2—C6	−156.2 (3)	Mn1—O4—C18—C19	176.3 (3)
O2—Mn1—N2—C6	134.0 (2)	Mn1—O3—C18—O4	5.1 (3)
C18—Mn1—N2—C6	−130.9 (2)	Mn1—O3—C18—C19	−176.0 (3)
O1—Mn1—N3—C15	76.8 (3)	O1—Mn1—C18—O4	22.7 (3)
O3—Mn1—N3—C15	−82.5 (3)	O3—Mn1—C18—O4	−175.2 (3)
N1—Mn1—N3—C15	176.5 (3)	N1—Mn1—C18—O4	−133.2 (2)
N2—Mn1—N3—C15	170.3 (2)	N2—Mn1—C18—O4	−64.1 (2)
O4—Mn1—N3—C15	−45.3 (3)	N3—Mn1—C18—O4	147.8 (2)
O2—Mn1—N3—C15	23.1 (3)	O2—Mn1—C18—O4	66.4 (2)
C18—Mn1—N3—C15	−65.4 (3)	O1—Mn1—C18—O3	−162.16 (17)
O1—Mn1—N3—C11	−110.7 (2)	N1—Mn1—C18—O3	42.0 (2)
O3—Mn1—N3—C11	90.1 (2)	N2—Mn1—C18—O3	111.04 (19)
N1—Mn1—N3—C11	−11.0 (2)	N3—Mn1—C18—O3	−37.0 (2)
N2—Mn1—N3—C11	−17.1 (3)	O4—Mn1—C18—O3	175.2 (3)
O4—Mn1—N3—C11	127.2 (2)	O2—Mn1—C18—O3	−118.41 (19)

### Hydrogen-bond geometry (Å, °)

**Table:**

*D*—H···*A*	*D*—H	H···*A*	*D*···*A*	*D*—H···*A*
O5—H5A···O6^i^	0.83 (1)	2.02 (2)	2.840 (4)	168 (5)
O5—H5B···O1^ii^	0.84 (1)	1.99 (2)	2.815 (4)	168 (5)
O6—H6A···O3	0.84 (1)	2.00 (1)	2.841 (4)	174 (5)
O6—H6B···O2^ii^	0.85 (1)	2.05 (2)	2.879 (4)	168 (5)

Symmetry codes: (i) *x*, −*y*+1/2, *z*+1/2; (ii) *x*, −*y*+1/2, *z*−1/2.
